# Palmitoylation at Two Cysteine Clusters on the C-Terminus of GluN2A and GluN2B Differentially Control Synaptic Targeting of NMDA Receptors

**DOI:** 10.1371/journal.pone.0049089

**Published:** 2012-11-15

**Authors:** Hayley A. Mattison, Takashi Hayashi, Andres Barria

**Affiliations:** 1 Department of Physiology and Biophysics, University of Washington School of Medicine, Seattle, Washington, United States of America; 2 Department of Molecular Neurobiology and Pharmacology, Graduate School of Medicine, University of Tokyo, Tokyo, Japan; Institute for Interdisciplinary Neuroscience, France

## Abstract

Palmitoylation of NMDARs occurs at two distinct cysteine clusters in the carboxyl-terminus of GluN2A and GluN2B subunits that differentially regulates retention in the Golgi apparatus and surface expression of NMDARs. Mutations of palmitoylatable cysteine residues in the membrane-proximal cluster to non-palmitoylatable serines leads to a reduction in the surface expression of recombinant NMDARs via enhanced internalization of the receptors. Mutations in a cluster of cysteines in the middle of the carboxyl-terminus of GluN2A and GluN2B, leads to an increase in the surface expression of NMDARs via an increase in post-Golgi trafficking. Using a quantitative electrophysiological assay, we investigated whether palmitoylation of GluN2 subunits and the differential regulation of surface expression affect functional synaptic incorporation of NMDARs. We show that a reduction in surface expression due to mutations in the membrane-proximal cluster translates to a reduction in synaptic expression of NMDARs. However, increased surface expression induced by mutations in the cluster of cysteines that regulates post-Golgi trafficking of NMDARs does not increase the synaptic pool of NMDA receptors, indicating that the number of synaptic receptors is tightly regulated.

## Introduction

The activation of NMDA-type glutamate receptors (NMDARs) promotes a wide range of signaling pathways that underlie the development, maturation, plasticity, and elimination of synapses in the central nervous system [1], [2]. NMDARs are composed of an obligatory GluN1 subunit, and one or more of the GluN2 subunits (A-D). All glutamate receptor subunits are composed of four domains: extracellular amino-terminal domain (NTD), extracellular ligand binding domain (LBD), trans-membrane domain (TMD), and intracellular carboxyl-terminal domain (CTD) [3]. The subunit composition of NMDARs determines the biophysical and pharmacological properties of the receptor. In the mammalian forebrain, the subunits most commonly expressed are GluN2A and GluN2B that occur as GluN1/GluN2 assemblies [4], [5].

Glutamate receptors are synthesized in the somatic endoplasmic reticulum and processed in the Golgi apparatus [5], [6]. They undergo several forms of post-translational modifications, phosphorylation being the most studied. Other post-translational modifications like palmitoylation and glycosylation have been described [3], although their role in receptor trafficking has only recently begun to be explored [7], [8]. NMDARs are synthesized in the somatic ER but sorted away from AMPA-type glutamate receptors (AMPARs), bypassing the somatic Golgi, and transported to dendritic Golgi outposts before being inserted into the plasma membrane [9].

Post-translational protein modifications of ionotropic glutamate receptors can regulate function, trafficking, protein-protein interactions, insertion into the plasma membrane, and synaptic targeting. For instance, phosphorylation of AMPARs plays a central role in synaptic plasticity by increasing the number of AMPARs at the synapse [10] and increasing single channel conductance [11], [12]. Phosphorylation of NMDARs also can regulate function, trafficking, surface expression, and internalization of these receptors [5], [13], [14], [15].

Palmitoylation is another type of reversible protein modification that regulates the surface trafficking of both AMPARs and NMDARs [7], [16]. Post-translational palmitoylation results from the addition of palmitic acid to proteins at intracellular cysteine residues [17]. Both GluN2A and GluN2B subunits have cysteine (Cys) clusters in the carboxyl-terminal domain that regulate trafficking of NMDARs [7].

The first cluster, Cys cluster I, is proximal to TMD IV and therefore close to the membrane. Palmitoylation of Cys cluster I stabilizes NMDARs in the membrane enhancing their surface expression. Stabilization of the receptor in the membrane increases tyrosine phosphorylation of an internalization motif, disrupting the interaction with adaptor protein AP-2 and therefore decreasing clathrin-mediated endocytosis. Thus, mutating three cysteines in the membrane-proximal cluster to non palmitoylatable serines (GluN2A 3CS and GluN2B 3CS) increases endocytosis, resulting in a reduction of NMDARs at the surface.

The second cluster, Cys cluster II, is in the middle of the carboxyl-terminus domain of GluN2 subunits. Palmitoylation of Cys cluster II promotes the accumulation of NMDARs in the Golgi apparatus, thereby reducing their surface expression [7]. Mutation of cysteines in this cluster to non-palmitoylatable serines (GluN2A 4CS and GluN2B 5CS) results in an increase in the surface expression of NMDARs [7].

Mutants of both Cys clusters have been characterized biochemically and their cellular distribution analyzed using fluorescence microscopy [7]. While it was determined that palmitoylation at these two clusters affects the surface expression of NMDARs, the functional synaptic expression of NMDARs containing these mutations was not assessed. We hypothesized that the expression of GluN2A 3CS and GluN2B 3CS would reduce the number of NMDARs at the synapse while the expression of GluN2A 4CS or GluN2B 5CS would increase the number of NMDARs at the synapse. We co-expressed mutant GluN2 subunits with a mutant of GluN1, GluN1 N598R that eliminates magnesium blockade and therefore the voltage-dependence of assembled NMDARs [18], [19]. This GluN1 mutant acts as an electrophysiological tag allowing detection of recombinant NMDARs when they are incorporated into synapses. This technique provides a quantitative method to analyze the level of synaptic incorporation of recombinant NMDARs in CA1 pyramidal neurons in hippocampal slices [20]. We found that the expression of GluN2A 3CS or GluN2B 3CS reduces the synaptic incorporation of NMDARs compared to wild type GluN2 subunits. However, expression of GluN2A 4CS or GluN2B 5CS did not increase synaptic incorporation of NMDARs relative to wild type GluN2 subunits, indicating that synaptic expression is more tightly regulated than surface expression.

## Results

We used a simple and quantitative assay to test whether palmitoylation of GluN2 subunits is necessary for functional incorporation of NMDARs into synapses.

**Figure 1:**
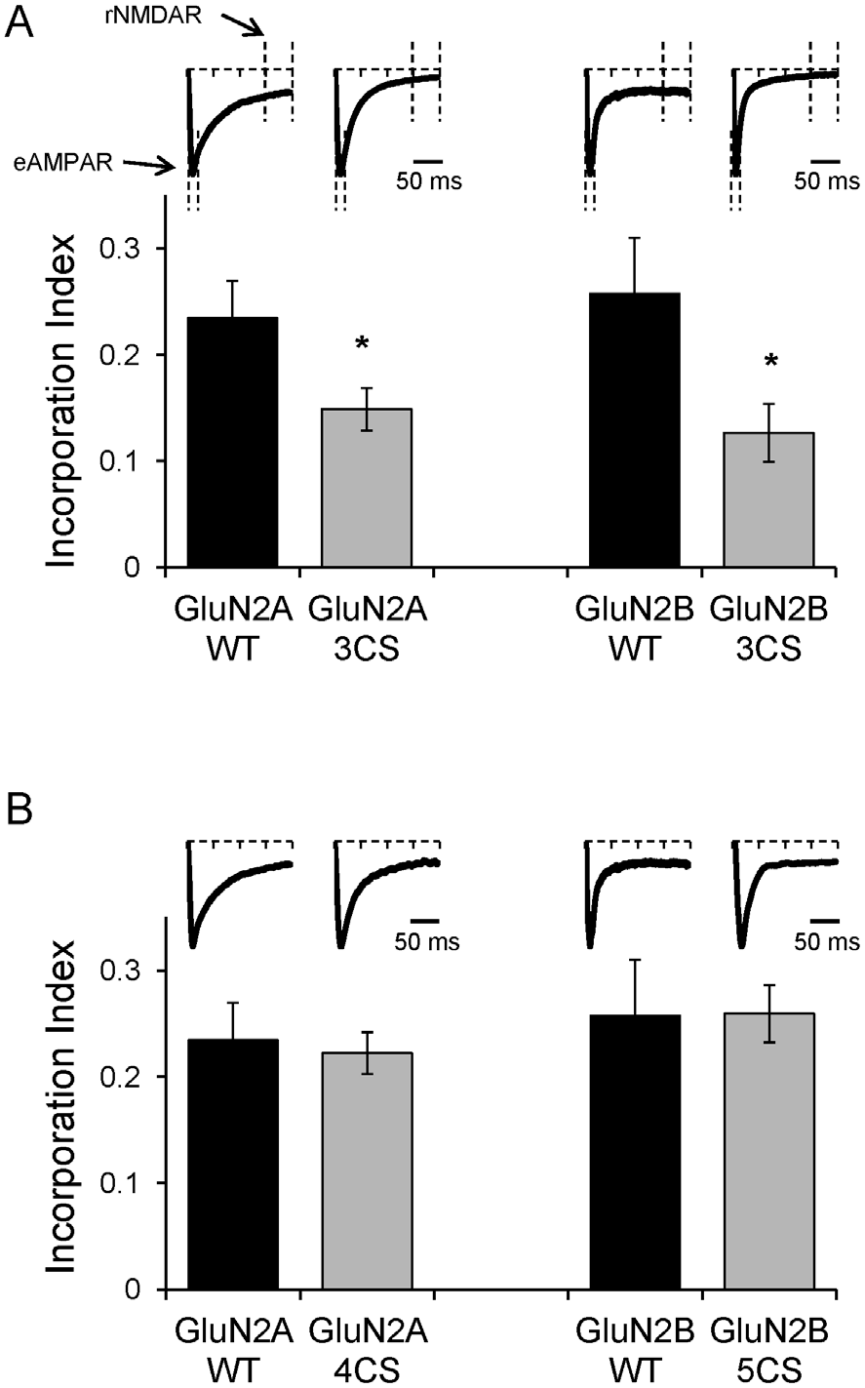
Incorporation Index of NMDARs containing GluN2 non-palmitoylatable mutants. **A.** Evoked EPSCs were recorded in CA1 pyramidal cells transfected with etag GluN1 and either wild type GluN2A (n = 9), GluN2A 3CS (n = 13), wild type GluN2B (n = 8), or GluN2B 3CS (n = 15). Evoked EPSCs were recorded at −70 mV and the Incorporation Index calculated as the ratio of the mean current from a 50 ms window 150 ms after the stimulus artifact corresponding to recombinant NMDARs (rNMDAR) normalized to the peak of the EPSC occurring within 50 ms from the stimulus artifact corresponding to endogenous AMPARs (eAMPAR). Error bars are s.e. Asterisk indicates p<0.05. Insets, example traces of EPSCS evoked in transfected cells as indicated. Vertical dotted lines indicate where measurements are taken. Scale bar = 50 ms **B.** In corporation Index measured as indicated in A from CA1 pyramidal cells transfected with etag GluN1 and either wild type GluN2A (n = 9), GluN2A 4CS (n = 15), wild type GluN2B (n = 8), or GluN2B 5CS (n = 18). Wild type values same as in A repeated here for easy comparison. Insets are example traces of EPSCs from cells transfected as indicated.

Cultured organotypic hippocampal slices were prepared from p6 rats and cultured for 4–5 days [21]. Using biolistics [22], slices were transfected with wild type GluN2 subunits or mutant GluN2 subunits where palmitoylated cysteines have been replaced with non palmitoylatable serines [7]. After transfection, slices were cultured for additional 3 days. GluN2 subunits were co-expressed with a mutant of GluN1 electrophysiologically tagged (etag GluN1). This tag allows monitoring functional incorporation of NMDARs into synapses [8], [20]. Briefly, mutant GluN1 N598R eliminates the normal magnesium blockade of NMDARs observed at hyperpolarized potentials [19], [23]. NMDAR subunits were also optically tagged with GFP to identify transfected CA1 neurons. Transfected cells expressing etag GluN1 and GFP-GluN2 subunits exhibit evoked excitatory post synaptic currents (EPSCs) at hyperpolarized potentials (−70 mV) with a fast component due to the activation of endogenous AMPARs and a slow component that reflects the activation of recombinant NMDARs (Fig. 1, Insets). Previously reported data show that etag GluN1 does not heteromerize with endogenous GluN2 subunits; therefore it is inserted into synapses only when heteromerizes with recombinant GluN2 subunits [20]. Because endogenous NMDARs are blocked by magnesium at −70 mV, the late component of evoked EPSCs at −70 mV in transfected neurons is measured to monitor whether recombinant NMDARs have been incorporated into synapses. An incorporation index was calculated in order to normalize the amount of current carried by recombinant NMDARs and allow cross cell comparisons; we measured a 50 milliseconds window in the late component of the EPSC and normalize it to the peak amplitude of the early component that correspond to the activation of endogenous AMPARs (Figure 1, insets).

While GluN1 subunits of the NMDAR are not palmitoylated, two conserved clusters of cysteines, Cys clusters I and II, have been described in both GluN2A and GluN2B subunits of NMDARs [7].

Cys cluster I is located immediately after the last transmembrane domain of GluN2 subunits in a position adjacent to the membrane in the carboxyl-terminus domain of GluN2A and GluN2B subunits. Palmitoylation of Cys cluster I increases the surface expression of NMDARs.

A second Cys cluster, Cys cluster II, is located in the middle of the carboxyl-terminus domain of GluN2 subunits. This cluster contains 4 Cys residues that are palmitoylated in GluN2A and 5 Cys residues in GluN2B. Palmitoylation of cluster II is catalyzed by palmitoyl acyl transferase (PAT) GODZ and causes accumulation of the receptor in the Golgi apparatus. Thus, palmitoylation of Cluster II decreases the surface expression of NMDARs.

### Palmitoylation of Cys Cluster I is Necessary for Functional Synaptic Incorporation of NMDARs

Mutations that prevent palmitoylation of cluster I either on GluN2A or GluN2B reduce the surface expression of NMDARs by increasing the rate of constitutive internalization of NMDARs [7]. First we asked whether these non-palmitoylatable GluN2 subunits can be incorporated into synapses at a normal rate despite the low overall surface expression.

Cultured organotypic hippocampal slices were co-transfected with etag GluN1 and wilt type GFP-GluN2 or mutant GFP-GluN2 subunits in which all 3 cysteine residues of cluster I have been replaced with non-palmitoylatable serines (GluN2A 3CS and GluN2B 3CS) [7]. Transfected CA1 pyramidal neurons were visualized and selected using fluorescence microscopy. Synaptic currents in CA1 pyramidal neurons were evoked by stimulation of Schaffer collateral fibers using a bipolar electrode and the Incorporation Index was measured.

The incorporation index for GluN2A wild type and GluN2B wild type, used here as controls, is similar to values previously reported [20], [24]. Synaptic incorporation of NMDARs containing GluN2A 3CS or GluN2B 3CS was significantly lower than the control wild types (Figure 1A) as can be seen in a decrease in the late component of evoked EPSCs (Figure 1A, insets). Thus, a reduction in the surface expression of NMDARs is translated in a reduced incorporation of NMDARs into synapses.

### Palmitoylation of Cys Cluster II does not Regulate Functional Synaptic Incorporation of NMDARs into Synapses

Palmitoylation of Cys cluster II has been shown to increase retention of newly synthesized NMDARs in the Golgi apparatus; therefore expression of non-palmitoylatable mutants increases the surface expression of NMDARs [7]. Next, we tested whether an increase in surface expression of NMDARs translates to an increase in functional synaptic NMDARs.

Experiments were carried out in CA1 neurons co-expressing etag GluN1 and GluN2 mutants whose Cys residues in Cluster II have been replaced by non-palmitoylatable serines (GluN2A 4CS and GluN2B 5CS) and the Incorporation Index measured as described before.

Surprisingly, the incorporation index of both of these mutants is similar to the incorporation index of wild type GluN2 subunits (Figure 1B), indicating that an increase in surface expression of NMDARs does not translate to a larger number of synaptic NMDARs.

### Effects of Non-Palmitoylatable GluN2 Subunits on Glutamatergic Synaptic Transmission

Because expression of different GluN2 subunits could affect regular glutamatergic synaptic transmission, next we tested whether the non-palmitoylatable GluN2 subunits have an effect on endogenous AMPAR-mediated synaptic transmission as well as on NMDAR-mediated synaptic transmission.

We recorded evoked EPSCs from CA1 neurons co-expressing GluN2 mutants and wild type GluN1, i.e. without the electrophysiological tag. In order to study whether expression of GluN2 Cys clusters mutants affect normal glutamatergic transmission, we compared the amplitude of evoked EPSCs recorded under the same stimulation conditions from a transfected cell and an adjacent non-transfected cell (paired recordings). EPSCs were evoked by stimulating Schaffer collaterals with a bipolar electrode. Recording from two adjacent cells receiving the same stimulation eliminates differences in amplitude due to stimulation conditions [25], [26].

It has been shown that early overexpression of wild type GluN2A reduces AMPAR and NMDAR mediated synaptic transmission [20], while overexpression of wild type GluN2B has no effect on glutamatergic synaptic transmission [20], [24], [27].

Hippocampal slices were co-transfected with wild type GluN1 and GluN2 mutants. Schaffer collaterals were stimulated and the peak amplitude of EPSCs at −70 mV and +40 mV were measured. EPSCs at −70 mV are AMPAR mediated currents while EPSCs at +40 are NMDAR mediated currents. Peak amplitudes of transfected cells were compared with those of an adjacent non-transfected neuron that serves as a control (pair recordings [26]).

**Figure 2:**
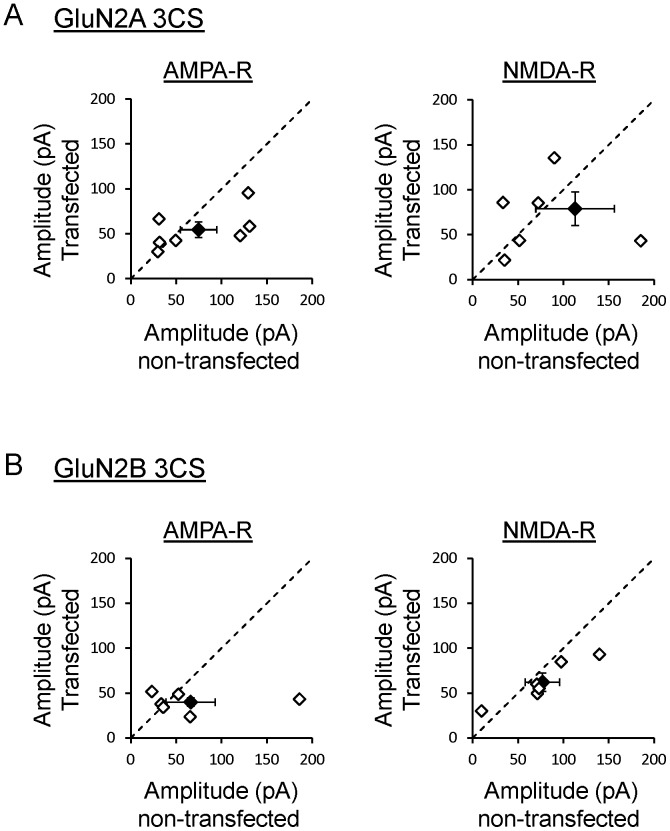
Pair recordings of AMPAR and NMDAR mediated EPSCs from neurons transfected with wild type GluN1 and GluN2 Cys cluster I mutants. A. Evoked responses recorded at −70 mV (AMPAR) or +40 mV (NMDAR) from neurons transfected with wild type GluN1 and GluN2A 3CS. Responses were evoked by stimulation of Schaffer collaterals and compared with an adjacent non-transfected neuron stimulated under the same conditions. Each dot represents a pair of neurons (n = 7 pairs). Black dot is average ± s.e. Dotted line is the unity line. **B.** Evoked responses from cells transfected with GluN2B 3CS recorded at −70 mV (AMPAR) or +40 mV (NMDAR). Responses from adjacent non-transfected neurons were recorded under the same stimulation conditions and compared with responses from transfected cells (n = 6 pairs).

Neurons transfected with GluN1 wild type and GluN2A 3CS exhibited a small decrease in AMPAR- as well as NMDAR-mediated synaptic transmission (Fig. 2A). Although this decrease is not statistically significant, this reduction is consistent with previous reports indicating that early expression of GluN2A subunits prevents normal synaptogenesis, therefore decreasing both AMPAR- and NMDAR-mediated synaptic transmission [28]. The reduction in AMPAR and NMDAR currents is 27% and 30% respectively (n = 7 pairs). This effect is smaller than the effect on synaptic transmission previously reported [28] and is attributed to the fact that this mutant of GluN2A does not incorporate into synapses as well as wild type GluN2A (Figure 1A).

Expression of wild type GluN1 and GluN2B 3CS also produces a non-significant decrease in glutamatergic synaptic transmission mediated by AMPARs or NMDARs (Figure 2B). The reduction in AMPAR and NMDAR currents is 40% and 19% respectively (n = 6 pairs). This reduction in synaptic transmission is consistent with a reduction of surface GluN2B [7] and the necessary role of GluN2B for proper synaptogenesis and therefore normal levels of glutamatergic synaptic transmission [28].

**Figure 3:**
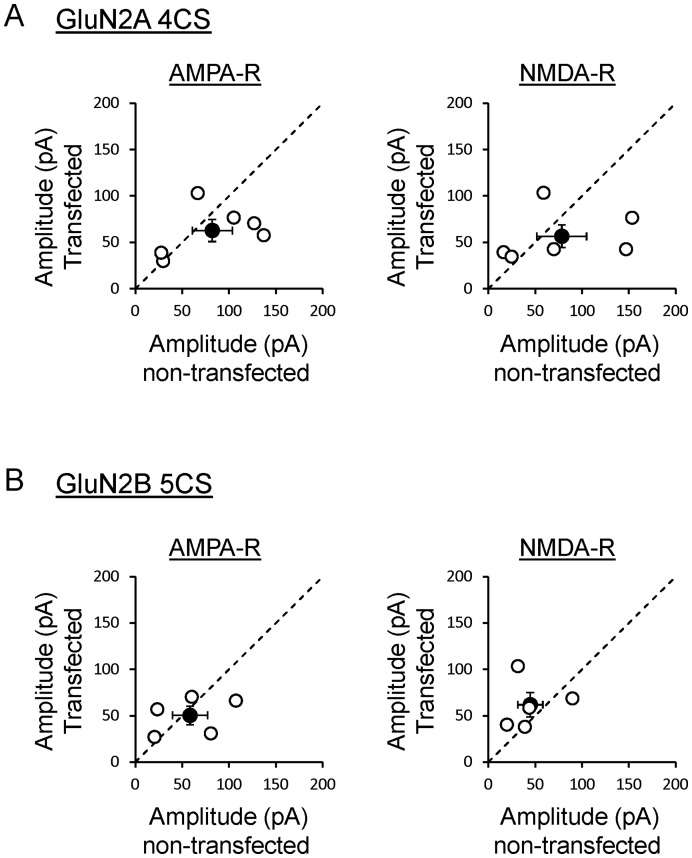
Pair recordings of AMPAR and NMDAR mediated EPSCs from neurons transfected with wild type GluN1 and GluN2 Cys cluster II mutants. A. Evoked responses recorded at −70 mV (AMPAR) or +40 mV (NMDAR) from neurons transfected with wild type GluN1 and GluN2A 4CS. Responses were evoked by stimulation of Schaffer collaterals and compared with an adjacent non-transfected neuron stimulated under the same conditions. Each dot represents a pair of neurons (n = 6 pairs). Black dot is average ± s.e. Dotted line is the unity line. **B.** Evoked responses from cells transfected with GluN2B 5CS recorded at −70 mV (AMPAR) or +40 mV (NMDAR). Responses from adjacent non-transfected neurons were recorded under the same stimulation conditions and compared with responses from transfected cells (n = 5 pairs).

Interestingly, expression of GluN2A mutant where Cys cluster II has been replaced with serines affects only slightly synaptic transmission (Figure 3A). GluN2A 4CS increases surface expression of NMDARs [7] and incorporates into synapses at levels similar to wild type GluN2A (Fig. 1B), however it does not reduce synaptic transmission as strongly as wild type GluN2A [24], [28]. The reduction in AMPAR and NMDAR currents is 24% and 28% respectively (n = 6 pairs) and does not reach statistical significance.

Expression of GluN2B mutant with Cys cluster II mutated to serines (GluN2B 5CS) does not change either AMPAR- or NMDAR-mediated synaptic transmission (Figure 3B) despite the fact that this mutant increases surface level of NMDARs [7]. This is consistent with previous reports showing that overexpression of GluN2B containing receptors does not alter glutamatergic synaptic transmission [20], [24], [27].

## Discussion

Palmitoylation of NMDAR subunits GluN2A and GluN2B occurs at 2 distinct Cys clusters and is a reversible post-translational modification that differentially regulates the surface expression of NMDARs [7]. Mutations in Cys cluster I (GluN2A 3CS, GluN2B 3CS) reduce the surface expression of NMDARs via an increase in the constitutive internalization of NMDARs. Mutations in Cys cluster II (GluN2A 4CS, GluN2B 5CS), on the other hand, enhance the surface expression of NMDARs by removing a Golgi retention signal and allowing forward trafficking of the receptor.

Reduction of surface expression of NMDARs via mutations in Cys cluster I also reduce synaptic incorporation of NMDARs suggesting that the increased internalization observed previously [7] also affects the number of NMDARs at the synapse. Because GluN2B-containing receptors are inserted into synapses in a constitutive manner [20] presumably via lateral diffusion [29], a reduction in the overall number of surface GluN2B containing receptors will indeed affect the availability of the receptor to be incorporated into synapses. Synaptic incorporation of GluN2A-containing receptors requires synaptic activity and occurs via replacement of existing synaptic GluN2B containing receptors [20], [30], [31]. Thus, a decrease in the synaptic incorporation of non-palmitoylatable GluN2A mutants could imply that their removal occurs from synaptic sites. However, we cannot rule out that mutant GluN2A receptors are incorporated extra-synaptically prior to removal, therefore diminishing the available pool of receptors that could incorporate into synapses.

Interestingly, expression of both non-palmitoylatable GluN2 subunits at Cys cluster I reduces endogenous synaptic transmission. Although no statistical significance is reached, this effect is in the same direction that previous observations. Overexpression of GluN2A in cultured hippocampal slices produces a dramatic reduction in AMPAR- and NMDAR-mediated synaptic transmission [20], [24], [28]. This has been attributed to the failure of GluN2A to interact with CaMKII and the prevention of synaptogenesis during the 3 day expression period [28]. Thus, a mutant of GluN2A that does not incorporate into synapses efficiently is expected to have a less profound effect on synaptic transmission. On the other hand, it has been shown that GluN2B is necessary and sufficient to allow proper synaptogenesis because it promotes spine motility and, presumably, the establishment of new synaptic connections [28]. Experiments in which GluN2B is knocked down after the initial period of synaptogenesis reduces the number of synapses formed [28]. Similarly, the small reduction in synaptic transmission reported here could be explained by a deficit of surface GluN2B receptors necessary for proper synaptogenesis.

A small reduction in AMPAR mediated synaptic transmission caused by expression of Cys cluster I mutants (Fig. 2A and B, left panels) could artificially overestimate the synaptic incorporation index of these mutants. However, the synaptic incorporation index of Cys cluster I mutants is very low and significantly different from wild type GluN2 subunits. Similarly, the reduction in AMPAR mediated synaptic transmission observed when Cys cluster II mutants are expressed is small or nonexistent (Fig. 3 A and B, left panels); therefore the synaptic incorporation index of these mutants is left unaltered and remains very robust, similar to wild type (Fig. 1B).

A surprising result is the fact that Cys cluster II mutants do not exhibit an increase in synaptic incorporation compared to wild-type NMDARs. These mutants exhibited enhanced surface expression because they are not retained in the Golgi apparatus; thus, we expected to see a larger incorporation of Cys cluster II mutant receptors than wild type receptors. However, Cys cluster II mutants incorporate into synapses as well as wild-type NMDARs Thus, increasing the pool of extrasynaptic receptors does not lead to an increase in the synaptic pool, indicating that the size of the synaptic pool is tightly regulated, possibly by limitations in the availability of interacting PDZ proteins [2].

It has been shown that overexpression of GluN2B containing receptors *in vitro* and *in vivo* does not increase NMDAR or AMPAR mediated synaptic transmission [20], [24], [27]. Consistent with this, expression of the GluN2B Cys cluster II mutant does not affect synaptic transmission.

In contrast, it has been shown that expression of GluN2A reduces synaptic transmission by reducing the number of synapses formed during the expression period [28]; thus, we expected to observe a similar reduction in AMPAR- and NMDAR-mediated currents with the expression of non-palmitoylatable GluN2A. However, only a small non-statistically significant effect was observed. This suggests that the Cys cluster II mutant of GluN2A, despite that incorporates into synapses at the same level as wild type GluN2A does not prevent the normal development of synapses as its wild type counterpart does. Since this cluster lies in the middle of the carboxyl-terminus of GluN2 subunits [7], it is possible that these mutations increase the interaction of GluN2Awith CaMKII thus reversing the negative effect of GluN2A on synapse number. Alternatively, it has been suggested that GluN2A could recruit to synapses negative regulators of synaptic plasticity [32], thus, mutations here could affect these interactions as well.

Palmitoylation is likely to play a central role in the trafficking of ionotropic glutamate receptors, and has already been shown to regulate surface trafficking of both AMPARs and NMDARs [7], [16], [33].We demonstrate that while surface expression is regulated by palmitoylation, other factors have a role in determining the number of NMDARs at the synapse. Lateral diffusion seems to deliver receptors from extrasynaptic pools to synapses because two mutants that reduce surface NMDARs, GluN2A 3CS and GluN2B 3CS, also reduce their presence at synapses. Importantly, a larger extrasynaptic pool does not translate to a larger synaptic pool, indicating that synaptic expression is tightly regulated. NMDARs are incorporated into synapses to match the level of synaptic activity [34]; therefore, an increase in the number of surface receptors will not necessarily drive more receptors into synapses unless synaptic activity is reduced.

Evidence indicates that trafficking of GluN2B subunits is constitutive [20] and that they are present on the surface of neurons before synapses are formed [35], with palmitoylation increasing their surface stability or Golgi retention [7]. On the other hand, GluN2A seems to be trafficked directly to spines and requires activity to be incorporated at synapses [20]; however the precise site of insertion into the plasmatic membrane of NMDARs with different GluN2 subunits is not known.

Further studies are needed to investigate the mechanisms that regulate the activity-dependent synaptic insertion of NMDARs, and how palmitoylation regulates the proteins that interact with NMDARs along the trafficking pathway.

## Methods

### Slice Cultures and Transfection

Organotypic hippocampal slices were prepared according to University of Washington guidelines from 6 day old (p6) Sprague Dawley male and female rats as described previously [21]. Animals were decapitated and tissue harvested according to protocol approved by UW Institutional Animal Care and Use Committee (IACUC).

After 3–5 days in culture, slices were transfected using a biolistic particle delivery system [22] and cultured for an additional 48–72 h. A plasmid DNA, ratio 1∶1 of GluN1 and GluN2 subunits was used (70–100 µg each).

### Constructs

GFP-GluN2A and GFP-GluN2B with mutations at C-terminal Cys clusters I and II were prepared as described before [7]. Membrane proximal cluster I mutants are notated as GluN2A 3CS and GluN2B 3CS and cluster II mutants in the middle of C-terminus are notated as GluN2A 4CS and GluN2B 5CS, with the numbers preceding CS indicating the number of cysteine residues mutated in each cluster.

### Electrophysiology

Whole-cell recordings from CA1 neurons were performed under visual guidance. The recording chamber was perfused with artificial CSF (ACSF) containing the following: 119 mM NaCl, 2.5 mM KCl, 4 mM CaCl2, 4 mM MgCl2, 26 mM NaHCO3, 1 mM NaH2PO4, 11 mM glucose, 100 µM picrotoxin (Tocris Bioscience), 2 µM 2-chloroadenosine, pH 7.4, gassed with 5%CO2/95%O2 at room temperature (20–25°C). Intracellular recording solution contained the following (in mM): 115 cesium methanesulfonate, 20 CsCl, 10 HEPES, 2.5 MgCl2, 2 MgATP, 2 Na2ATP, 0.4 Na3GTP, 10 sodium phosphocreatine, 5 QX-314, and 0.6 EGTA (pH 7.25 and 310 mmol/Kg). Synaptic responses were evoked with bipolar cluster electrodes (FHC) placed over Schaffer collateral fibers. The synaptic incorporation index is calculated from the mean current from a 50 ms window 150 ms after the stimulus artifact in evoked EPSCs recorded at −70 mV corresponding to the recombinant NMDAR, normalized to the peak of the EPSC occurring within 50 ms from the stimulus artifact corresponding to the activation of endogenous AMPARs.
